# Effect of sodium balance on levels of nitrates in healthy subjects: Posthoc analysis of a randomized, double‐blinded, placebo‐controlled, crossover study

**DOI:** 10.14814/phy2.70512

**Published:** 2025-08-19

**Authors:** A. M. Østergaard, R. L. Sønderbæk, M. H. Vrist, J. B. Rosenbæk, F. H. Mose, J. N. Bech

**Affiliations:** ^1^ Department of Medicine, University Clinic in Nephrology and Hypertension Gødstrup Hospital Herning Denmark; ^2^ Department of Clinical Medicine Aarhus University Aarhus Denmark

**Keywords:** blood pressure, glomerular filtration rate, nitrate, nitric oxide, nitrite, sodium excretion

## Abstract

Nitric oxide (NO) regulates renal sodium handling, but the relationship between sodium intake, NO synthesis, and nitrate/nitrite levels is unclear in humans. In a randomized, placebo‐controlled, double‐blinded, crossover study, 27 healthy subjects followed a 4‐day low‐sodium diet with either sodium chloride tablets or placebo daily, separated by a 3‐week washout. Blood pressure, pulse wave velocity, and glomerular filtration rate were assessed. Blood and 24‐h urine samples were analyzed for nitrate, nitrite, cyclic guanosine monophosphate (cGMP), and electrolytes. Plasma nitrate levels were lower after high sodium intake (*p* = 0.019), while plasma nitrite and cGMP levels remained unchanged. Urinary excretion of nitrate and nitrite did not differ, but nitrate clearance and fractional excretion increased (both *p* = 0.010). Renal handling of nitrite was unchanged. No significant differences were observed in blood pressure, pulse wave velocity, or glomerular filtration rate. When stratified by sex, females showed higher plasma nitrate after low sodium and a decrease after high sodium. Females decreased urinary nitrate excretion, whereas males remained stable. In conclusion, high sodium intake decreased plasma nitrate without increased excretion, suggesting greater nitrate utilization, particularly in females. ClinicalTrials.gov Identifier: NCT06968182.

## 1 INTRODUCTION

Nitric oxide (NO) is a short‐lived molecule with numerous physiological roles, including regulation of systemic vascular tone and cardiovascular health, as well as renal water and sodium homeostasis (Bech et al., 2007; Kurtz & Wagner, 1998; Tousoulis et al., 2012). The formation of NO is an oxygen‐dependent process initiated by the amino acid L‐arginine and catalyzed by nitric oxide synthase (NOS), known as the classical pathway (Lundberg & Weitzberg, 2009; Palmer et al., 1988). NO stimulates guanylate cyclase, which further activates cyclic guanosine monophosphate (cGMP), a key second messenger in NO‐mediated signaling (Denninger & Marletta, 1999). For years, inorganic nitrate and nitrite were considered biologically inert end products of classical NO synthesis (Lundberg et al., 2008). However, a reverse pathway has since been identified, in which nitrate is stepwise reduced to nitrite and subsequently to NO, known as the alternative pathway (Bailey & Dhaun, 2024; Kiraku et al., 1999; Larsen et al., 2012; Tzemos et al., 2008). Excess sodium intake is a major independent risk factor for cardiovascular disease, including hypertension (He et al., 2020; Jaques et al., 2021). NO has been suggested to play a role in renal adaptation to increased sodium intake (Abe et al., 2006; Atucha et al., 1994; Bailey & Dhaun, 2024; Bech et al., 1998, 2007; Larsen et al., 2012; Romero & Strick, 1993). Accordingly, several studies have demonstrated increased NO synthesis following high sodium intake (Chen & Sanders, 1993; Kiraku et al., 1999; Tzemos et al., 2008). Similarly, NOS inhibition has been shown to increase renal vasoconstriction and reduce natriuresis—effects that are further amplified under conditions of high sodium intake (Deng et al., 1994, 1995; Tolins & Shultz, 1994). Additionally, increased urinary nitrate and nitrite excretion has been associated with lower blood pressure (BP) in sodium‐loaded individuals (Smallwood et al., 2017).

Despite growing evidence for the alternative pathway, few studies have investigated the renal and tubular handling of inorganic nitrate and nitrite in humans (Kapil et al., 2018; Sundqvist et al., 2021). Interestingly, existing data have revealed significant sex differences in plasma levels and renal handling of nitrate and nitrite, both before and after supplementation. However, the influence of controlled sodium balance on plasma and urinary nitrate/nitrite concentrations has not previously been investigated. In the present study, we aimed to investigate the effects of 4 days of low versus high sodium intake on plasma and urinary levels of nitrate and nitrite, as well as hemodynamic parameters, in healthy subjects. Based on the previously mentioned findings, we hypothesized that sodium loading would increase plasma nitrate/nitrite levels and that sex‐related differences might be observed.

## 2 MATERIALS AND METHODS

### 2.1 Design

The study was a randomized, placebo‐controlled, double‐blinded, crossover study in 27 healthy subjects. The subjects received 4 days of a standardized, sodium‐reduced diet and either sodium chloride tablets or placebo in random order. Each dieting period was followed by an examination day, which was separated by at least 3 weeks' washout. The study was approved by the Regional Committee on Biomedical Research Ethics (case number: 1‐10‐72‐351‐15). Informed, signed consent was obtained from each subject. The study was carried out in accordance with the Declaration of Helsinki.

### 2.2 Recruitment

Subjects were recruited through announcements at public institutions and private companies. All subjects passed an examination before inclusion. The examination included medical history, physical examination, office BP measurement, ECG, urine analysis, and the following blood samples: Alanine aminotransferase, sodium, potassium, creatinine, albumin, platelets, leukocytes, and hemoglobin.

### 2.3 Subjects

#### 2.3.1 Inclusion criteria

Age 18–50 years, body mass index (BMI) 18.5–30.0 kg/m^2^.

#### 2.3.2 Exclusion criteria

Alcohol consumption >7 drinks per week for women and >14 drinks per week for men, office BP > 140/90, current smoking, drug abuse, medical treatment, pregnancy, nursing, neoplasia, anamnestic or clinical signs of significant disease, anemia, blood donated within the past month, and estimated glomerular filtration rate (eGFR) < 60 mL/min, and women of childbearing potential not using effective contraception throughout the examination period.

### 2.4 Withdrawal criteria

Withdrawal of consent, development of exclusion criteria, serious or unacceptable adverse events.

### 2.5 Outcomes

#### 2.5.1 Nitrogen oxides

Plasma levels of nitrate, nitrite, cGMP, urinary excretion and clearance of nitrate and nitrite, fractional excretion of nitrate (FE_nitrate_) and nitrite (FE_nitrite_).

#### 2.5.2 Water and salt regulation

GFR, urinary excretion of sodium (UNa), urinary excretion of potassium (UK), fractional excretion of sodium (FE_Na_), and urine output (UO).

#### 2.5.3 Hemodynamics

Brachial BP, heart rate and pulse wave velocity (PWV).

### 2.6 Calculations

Fractional excretions (FE) of nitrate or nitrite (X) were estimated by the formula FE_X_ = (Xu * V/Xp)/GFR. Xu and Xp are urine and plasma concentrations of X, and V is urine flow in mL/min.

Clearance of nitrate and nitrite was estimated by the formula Cl_X_ = (Xu*UV)/(Xp*UT) and body surface area adjusted, using the Du Bois formula.

Xu and Xp are urine and plasma concentrations of X; UV is the amount of urine in mL, and UT is the duration of the urine collection in minutes.

### 2.7 Number of subjects

This exploratory study is based on another randomized study (clinicaltrials.gov: NCT06968182). Therefore, no formal power calculation has been performed.

### 2.8 Randomization

The diet order was allocated by the Hospital Pharmacy, Central Denmark Region, Denmark. The randomization list was created according to a randomization plan created on “randomization.com”. All medication was packed, sealed, and labeled by the hospital pharmacy. Diet assignment and allocation were concealed from clinicians, patients, and research staff until completion of the trial.

### 2.9 Study medication

During the high sodium period, the subjects received 200 mmol sodium per day as 16 daily capsules. The sodium supplement was gelatin capsules, each containing three sodium chloride enterotablets 250 mg (cat.no: 249243, Medic, Viatris ApS, Denmark). The placebo supplement was gelatin capsules, each enclosing four tablets containing 86 mg potato starch and 85 mg lactose (cat.no: 802305, The Hospital Pharmacy of the Capital Region of Denmark). Gelatin capsules used for sodium supplement and placebo were identical in appearance (Capsugel, sweedish orange size AA, Lonza, Basel, Switzerland).

### 2.10 Experimental procedure

#### 2.10.1 Prior to examination

For 4 days prior to each examination day, subjects ingested a standard diet prepared by the hospital kitchen. According to the estimated energy demands for each subject based on weight and physical activity, a diet containing either 11,000 kJ or 15,000 kJ per day was chosen. The nutritional composition contained 55% carbohydrates, 30% fat, and 15% protein. Sodium content was approx. 100 mmol per day, and the content of nitrate and nitrite was minimized. Subjects were allowed to drink water as needed. A maximum of two small cups of coffee or tea was allowed, while no alcohol or soft drinks were permitted. The subjects were instructed to ingest the study medication three times daily between 7 and 8 am (6 capsules), between 12 am and 1 pm (5 capsules), and 6 and 8 pm (5 capsules), with the last dose the evening before the examination day.

#### 2.10.2 Examination day

On each examination day, the subjects arrived at the lab at 7:45 am after an overnight fast. The subjects were confined to a temperature‐controlled room (21–25°C) in a supine position throughout the examination day; voiding was done standing or sitting. Two intravenous catheters were placed into each antecubital vein, one for tracer infusion (^51^Cr‐EDTA) and one for blood sampling. To ensure hydration, subjects were given 175 mL of tap water every half hour from 7:30 am. Blood samples were collected at 8:30 am. ^51^Cr‐EDTA infusion was initiated at 9:00 am, and blood and urine samples analyzed for ^51^Cr‐EDTA were collected at 30 min intervals from 9:30 am to 11 am. These three clearance periods were pooled and used as GFR measurement. The blood samples collected at 8:30 am were analyzed for plasma levels of cGMP, nitrate, and nitrite. The urine sample collected at 9:30 am was further analyzed for sodium, potassium, nitrate, and nitrite.

### 2.11 Blood pressure and arterial stiffness measurements

Brachial BP, heart rate, and PWV were measured using the oscillometer Mobil‐O‐Graph® (I.E.M. GmbH, Aachen, Germany). The device was applied according to the directions of the manufacturer, and sequential measurements were made every half hour from 8:30 am to 11:00 am and estimated as the average of all measures.

### 2.12 Renal function

In this study, GFR was measured by the steady‐state clearance technique with ^51^Cr‐EDTA (GE Healthcare Limited, Chalfont Buckinghamshire, Great Britain) as the reference substance (Pedersen et al., 1984).

### 2.13 Biochemical analyses

Plasma and urine samples for potassium and sodium were analyzed at the Department of Clinical Biochemistry, Gødstrup Hospital, Denmark. Both plasma and urine samples for the analysis of cGMP were immediately placed in ice water and centrifuged at 1000G at 4°C for 15 min and frozen at −80°C until assayed. The samples were again centrifuged before being analyzed by a competitive enzyme immunoassay (cat.no: KGE003, R&D Systems, Minneapolis, USA). The minimal detection level was 1.14 pmol/mL. Coefficients of variation were 6.7% (intra‐assay) and 8.9% (interassay). All samples were analyzed with kits from the same batch. Plasma and urine samples of nitrate and nitrite were determined using the Zysense Nitric Oxide Analyser (NOA 280i) (Zysense, Frederick, Colorado, USA) via ozone chemiluminescence as previously described (Østergaard et al., 2023).

### 2.14 Statistical analysis

Values with a normal distribution are presented as means ± standard deviations (SD). Non‐normally distributed values are displayed as medians with interquartile ranges in brackets. Comparisons between diets were made using a paired *t*‐test when data were normally distributed and a Wilcoxon signed‐rank test when data were nonparametric. Comparisons between independent subgroups were made using an unpaired *t*‐test when data were normally distributed and the Mann–Whitney *U* test when data were nonparametric. A two‐way analysis of variance (ANOVA) was performed to evaluate the main effects of the intervention (low vs. high sodium), sex (male vs. female), and their interaction (intervention × sex) on all variables. Results are reported as *F*‐values, *p* values, and partial eta squared ($\eta_{p}^{2}$) to estimate effect sizes. Normality and homogeneity of variance were assessed prior to analysis. Determinations of correlations were completed using the Spearman correlation coefficient analysis. Statistical significance was defined as *p* < 0.05. Statistical analyses were performed using PASW version 20.0.0 (SPSS Inc., Chicago, IL, USA).

## 3 RESULTS

### 3.1 Demographics

**FIGURE 1:**
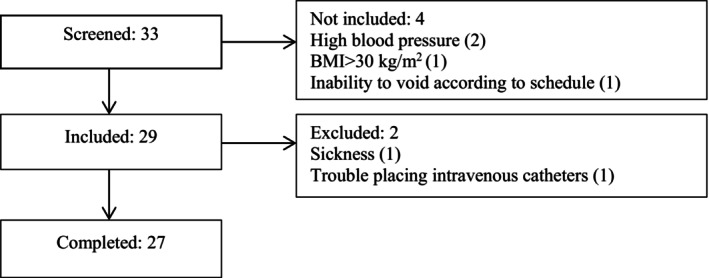
Flow diagram showing the flow through screening, inclusion, and completion of the trial.

Demographic characteristics are presented in Table 1. In total, 33 subjects were screened for eligibility. Of these, 29 were included, and 27 completed the study (Figure 1). Four subjects were excluded due to not meeting inclusion criteria: high BP (*n* = 2), BMI > 30 kg/m^2^ (*n* = 1), and inability to void according to schedule (*n* = 1). During the examinations, two were excluded due to sickness (*n* = 1) and trouble placing intravenous catheters (*n* = 1).

**TABLE 1:** Demographics (*n* = 27).

Male, % (*n*)	51% (14)
Age (years)	27 ± 7
Body mass index (BMI, kg/m^2^)	24 ± 2
Systolic blood pressure (mmHg)	122 ± 9
Diastolic blood pressure (mmHg)	70 ± 6
P‐creatinine (μmol/L)	76 ± 10
eGFR (mL/min)	109 ± 14
P‐potassium (mmol/L)	3.9 ± 0.3
P‐sodium (mmol/L)	139 ± 2
P‐albumin (g/L)	43.8 ± 2.7
B‐hemoglobin (mmol/L)	8.7 ± 0.8

*Note*: Values are shown as means ± SD.

### 3.2 The nitric oxide system

**FIGURE 2:**
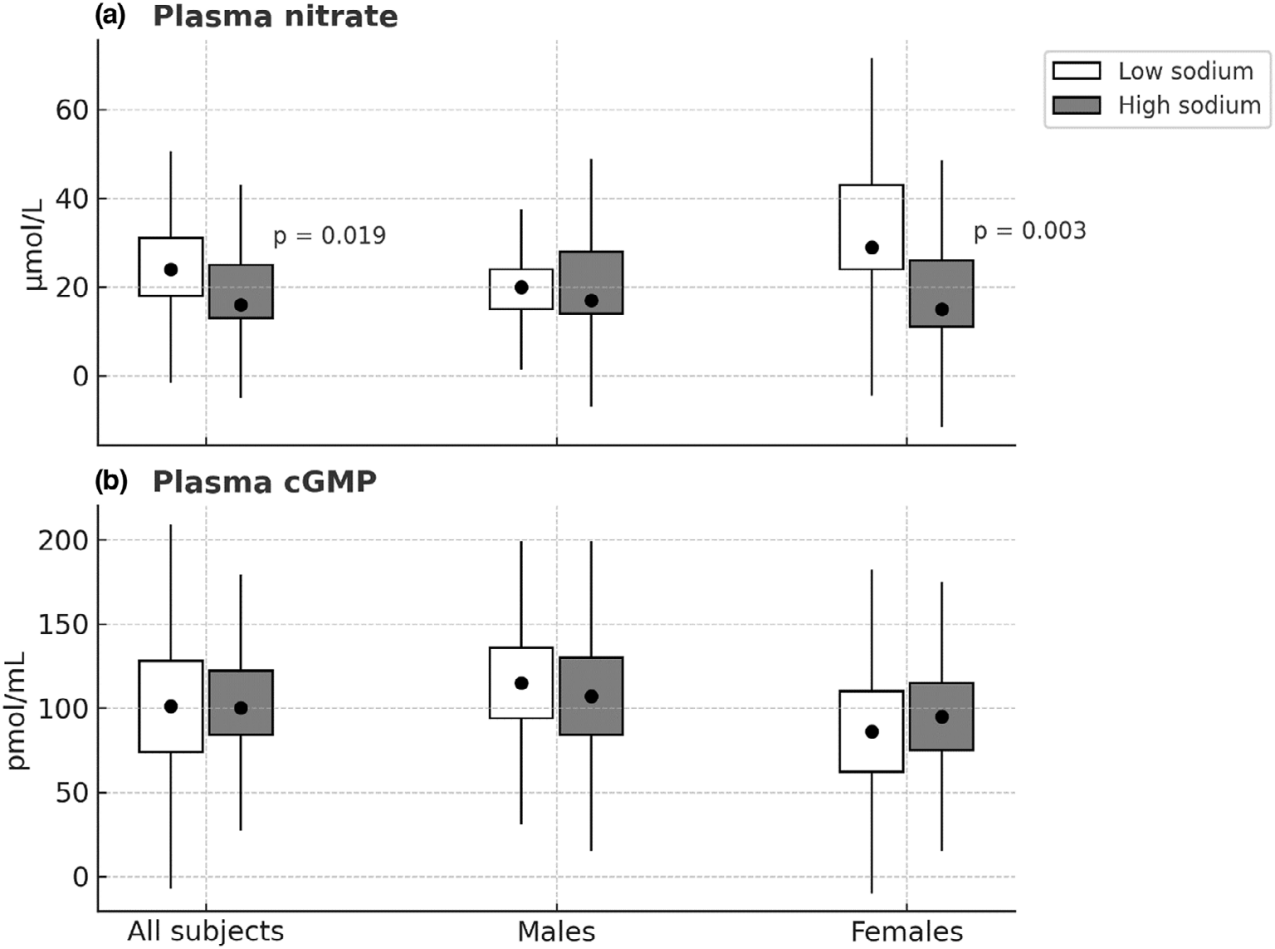
(a) Plasma concentrations of nitrate. (b) Plasma concentrations of cyclic guanosine monophosphate (cGMP). Data are from a randomized, crossover study of healthy subjects following 4 days of high or low sodium intak; all subjects (*n* = 27), males (*n* = 14), and females (*n* = 13). Data are presented as boxplots: (a) Represent median and interquartile range (IQR); whiskers indicate ±1.5 × IQR. (b) Represent mean ± standard deviation (SD); whiskers indicate ±1.5 × SD. Statistical comparisons between sodium conditions were performed using the Wilcoxon signed‐rank test for the overall group (All subjects), and the unpaired *t*‐test for comparisons within males and females, who represent independent subgroups.

All measured plasma and urinary levels of the nitric oxide system are shown in Table 2. Plasma nitrate (Figure 2) was significantly lower following high sodium intake compared with low sodium intake (*p* = 0.019). In contrast, plasma nitrite did not differ between diets.

**TABLE 2:** The nitric oxide system (*n* = 27).

Variable	Low sodium	High sodium	*p* Value
p‐Nitrate (μmol/L)	24 [18; 31]	16 [13; 25]	0.019
p‐Nitrite (μmol/L)	0.20 ± 0.06	0.22 ± 0.06	0.110
p‐cGMP (pmol/mL)	101 ± 27	100 ± 22	0.827
Nitrate/nitrite ratio plasma	118 [100; 178]	80 [53; 104]	0.002
U‐Nitrate (μmol/min)	0.96 [0.60; 1.20]	0.70 [0.47; 1.19]	0.290
U‐Nitrite (nmol/min)	1.4 [1.0; 1.8]	1.5 [1.1; 2.1]	0.130
Nitrate clearance (mL/min)	36 [31; 42]	40 [33; 49]	0.010
Nitrite clearance (mL/min)	7 [5; 9]	7 [4; 13]	0.414
Nitrate clearance (mL/min/m^2^)	19 [17; 22]	21 [18; 24]	0.011
Nitrite clearance (mL/min/m^2^)	4 [3; 5]	3 [2; 6]	0.471
FE_nitrate_ (%)	41 [36; 46]	45 [39; 51]	0.010
FE_nitrite_ (%)	8.5 [4.7; 10.5]	9.1 [4.4; 13.9]	0.374
Nitrate/nitrite ratio urine	678 [468; 984]	570 [295; 788]	0.032

*Note*: Plasma concentrations of nitrate, nitrite, cyclic guanosine monophosphate (cGMP) and the nitrate/nitrite ratio. Urinary excretion rate of nitrate (nitrate/minute), nitrite (nitrite/minute). Renal clearance of nitrate and nitrite (mL/minute), fractional excretion of nitrate (FE_nitrate_) and nitrite (FE_nitrite_) and the nitrate/nitrite ratio in urine. Values are shown as means ± SD in brackets or medians with 25 and 75 percentiles in brackets. Statistics are performed with a paired *t*‐test or Wilcoxon signed rank test to test difference in response between diets.

The plasma and urinary nitrate/nitrite ratios also changed significantly between diets (*p* = 0.002 and *p* = 0.032, respectively). However, plasma cGMP levels did not differ between diets (Figure 2).

### 3.3 Glomerular filtration rate

As expected, urinary sodium excretion was significantly higher after high sodium intake compared with low sodium intake (high: 286 ± 58 mmol/24 h vs. low: 101 ± 42 mmol/24 h; *p* < 0.001). Although not statistically significant, there was a trend toward higher glomerular filtration rate (GFR) following high sodium intake (high: 91 ± 14 mL/min/1.73 m^2^; low: 87 ± 14 mL/min/1.73 m^2^; *p* = 0.051). Urine output did not differ between diets (high: 7.9 ± 2.9 mL/min; low: 7.7 ± 3.4 mL/min; *p* = 0.745). Further data are provided in Table S1.

### 3.4 Systemic hemodynamic

Systolic and diastolic blood pressure, heart rate, and pulse wave velocity (PWV) did not differ significantly between diets. Full hemodynamic results are presented in Table S2, and data stratified by sex are provided in Table S3.

### 3.5 Correlations

The absolute changes from high to low sodium intake (Table 3) showed a weak positive correlation between plasma nitrite and both systolic blood pressure (Spearman's correlation, *R* ^2^ = 0.148, *p* = 0.047) and diastolic blood pressure (*R* ^2^ = 0.228, *p* = 0.012). These correlations are illustrated in Figures S1 and S2. No corresponding correlations were observed between changes in blood pressure and plasma nitrate, or between blood pressure and urinary nitrate/nitrite excretion (*p* > 0.05 for all correlations). These nonsignificant correlations are not shown. Furthermore, no correlations were found between the absolute changes in urinary sodium excretion and plasma nitrate or nitrite concentrations. No significant sex differences were observed in any of the correlation analyses.

**TABLE 3:** The absolute changes from high to low sodium in males (*n* = 14) and females (*n* = 13).

Variable	Men	Women	*p* Value
Δp‐Nitrate (μmol/L)	−0.60 ± 12.3	−14.8 ± 14.6	0.011
Δp‐Nitrite (μmol/L)	0.026 ± 0.070	0.019 ± 0.071	0.793
Δp‐cGMP (pmol/mL)	−10.0 ± 22.6	8.54 ± 25.2	0.055
ΔU‐Nitrate*V (μmol/min)	0.13 ± 0.34	−0.45 ± 0.54	0.004
ΔU‐Nitrite*V (nmol//min)	0.3 ± 0.68	0.2 ± 1.11	0.655
ΔNitrate clearance (mL/min)	5.6 [−11.9;0.95]	6.6 [−10.7;2.7]	0.943
ΔNitrite clearance (mL/min)	0.71 ± 7.4	0.15 ± 4.0	0.811
ΔNitrate clearance (mL/min/m^2^)	2.72 ± 4.0	1.74 ± 6.3	0.640
ΔNitrite clearance (mL/min/m^2^)	0.3820 ± 3.79	−0.0020 ± 2.28	0.722
ΔFE_nitrate_ (%)	5.9 [1,3;9.5]	3 [−4;10]	0.488
ΔFE_nitrite_ (%)	1.4 [−1,5;5.0]	−0.2 [−3.1;2.9]	0.430

*Note*: Plasma concentrations of nitrate, nitrite, and cyclic guanosine monophosphate (cGMP). Urinary excretion rate of nitrate (nitrate/minute) and nitrite (nitrite/minute). Renal clearance of nitrate and nitrite (mL/minute), fractional excretion of nitrate (FE_nitrate_), and nitrite (FE_nitrite_). Values are shown as means ± SD in brackets or medians with 25 and 75 percentiles in brackets. Statistics are performed with a unpaired *t*‐test or Mann–Whitney test to test difference in response between sex.

### 3.6 Effects of sodium intake, sex, and their interaction

**FIGURE 3:**
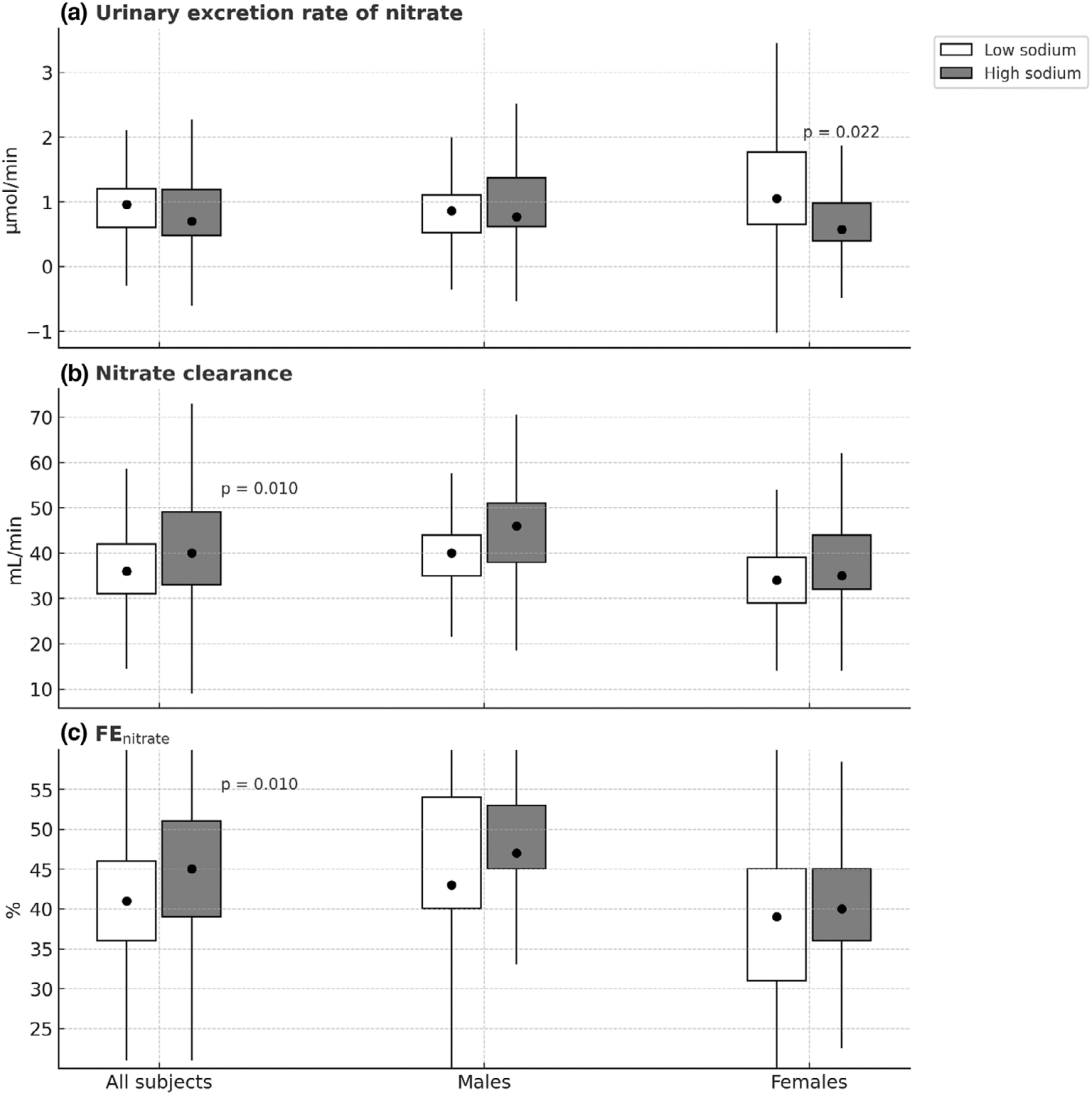
(a) Urinary excretion rate of nitrate. (b) Renal clearance of nitrate. (c) Fractional excretion of nitrate (FE_nitrate_). Data are from a randomized, cross‐over study of healthy subjects following 4 days of high or low sodium intake: All subjects (*n* = 27), males (*n* = 14), and females (*n* = 13). Data are presented as boxplots showing median and interquartile range (IQR); whiskers represent ±1.5 × IQR. Statistical comparisons between sodium conditions were performed using the Wilcoxon signed‐rank test for the overall group (All subjects), and the Mann–Whitney *U* test for comparisons within males and females, who represent independent subgroups.

Stratification of the dataset by sex (Figures 2 and 3, Tables S3 and S4) revealed that on a low sodium diet, plasma nitrate concentrations were significantly higher in females (29 [24; 43] μmol/L) than in males (20 [15; 24] μmol/L; *p* < 0.0001). Urinary nitrate excretion was also numerically higher in females (1.05 [0.65; 1.77] μmol/min) than in males (0.86 [0.52; 1.11] μmol/min), but this difference did not reach statistical significance (*p* = 0.15). Following high sodium intake, females exhibited a significantly greater reduction in both plasma nitrate concentrations (*p* = 0.003) and urinary nitrate excretion rates (*p* = 0.022) compared to low sodium intake. This pattern was not observed in males (Figures 2 and 3).

When comparing absolute changes from high to low sodium intake (Table 3), females showed a more pronounced decrease in plasma nitrate than males (*p* = 0.011); a similar sex difference was found for urinary nitrate excretion (*p* = 0.004), despite no detectable differences in GFR between sexes (Table S3). A trend toward a sex difference in the cGMP response to sodium intake was also observed (*p* = 0.055; Table 3).

Two‐way ANOVA confirmed significant main effects of sodium intake (low vs. high) on urinary sodium excretion and plasma nitrate concentrations. Significant main effects of sex were found for blood pressure, heart rate, plasma cGMP, and fractional nitrate excretion, indicating inherent physiological differences between males and females. Significant interaction effects between sodium intake and sex were identified for plasma nitrate (*F* (1, 50) = 6.914, *p* = 0.011, $\eta_{p}^{2}$ = 0.124) and urinary nitrate excretion (*F* (1, 50) = 6.113, *p* = 0.017, $\eta_{p}^{2}$ = 0.109). No other significant main or interaction effects were found. Full results are available in Table S5.

## 4 DISCUSSION

In this study, we found 4 days of high sodium intake in healthy subjects resulted in a lower level of plasma nitrate and modified the renal handling of nitrate with an increase in the renal clearance and the fractional excretion of nitrate.

Nitrate is known to be freely filtered by the glomerulus; however, its specific renal handling—and how this can be modulated—remains largely unidentified in humans. Our study population consisted of young, healthy individuals with an equal sex distribution. Thus, the findings during the period of low sodium intake can be considered baseline values representing unaltered renal nitrate handling. At baseline, we observed a nitrate clearance of 36 mL/min and a fractional excretion of 41%, suggesting that approximately 60% of filtered nitrate is reabsorbed. In comparison, Sundqvist et al. (2021) reported a lower nitrate clearance (females: 15 mL/min; males: 25 mL/min) and a lower fractional excretion (females: 16%; males: 21%) in a randomized, placebo‐controlled trial including 231 prehypertensive and hypertensive subjects (Sundqvist et al., 2021). Their findings suggest that up to 80% of filtered nitrate is reabsorbed. Several methodological differences may account for this discrepancy. First, the Sundqvist population was larger, but also older and either prehypertensive or hypertensive, and both age and hypertension have been associated with reduced NO bioavailability, which could influence renal handling (Lundberg et al., 2015). Second, the two studies used different analytical methods: we measured NOx via chemiluminescence, while Sundqvist et al. used high‐performance liquid chromatography (HPLC). Finally, we assessed GFR using the highly accurate steady‐state clearance of 51Cr‐EDTA, whereas Sundqvist estimated GFR from 24‐h creatinine clearance (Sundqvist et al., 2021). Contrary to our hypothesis, plasma nitrate or nitrite levels did not increase after high sodium intake. Instead, plasma nitrate was significantly lower following high compared to low sodium intake. Since urinary nitrate excretion did not increase, the mechanism underlying this reduction in plasma nitrate remains unclear. One possible explanation could be that high sodium intake does not enhance NO synthesis in humans, as suggested by previous experimental studies in rats. In fact, several human studies using forearm venous occlusion plethysmography and intra‐arterial infusions of acetylcholine (ACh) and NG‐monomethyl‐L‐arginine (L‐NMMA) have reported reduced vascular NO‐mediated vasodilation after high sodium intake (Bragulat et al., 2001; Lim et al., 2001; Miyoshi et al., 1997). These results indicate an impaired function of the classical L‐arginine–NO pathway following high sodium intake. Consequently, plasma nitrate levels may not reliably reflect total NO production under certain physiological conditions, such as high sodium intake. Thus, our findings of unchanged urinary nitrate excretion despite lower plasma nitrate levels may suggest an alteration in renal handling rather than a reduction in systemic synthesis. In humans, the specific renal handling of nitrate remains largely unidentified (Bryan et al., 2013; Godfrey & Majid, 1998; Majid et al., 1995). Thus, it is noteworthy that our findings of increased renal clearance and fractional excretion of nitrate following high sodium intake further support this interpretation. While these changes could theoretically result from enhanced tubular secretion or reduced tubular reabsorption, the stable GFR and total urinary nitrate excretion observed in our study indicate that increased systemic utilization of nitrate is more plausible. A theoretical explanation behind this response could involve activation of the alternative pathway. Namely, the reduction of nitrate to nitrite and subsequently to NO—as part of a compensatory mechanism to counteract a sodium induced rise in BP (Kapil et al., 2010; Rosenbæk et al., 2018). Several studies have demonstrated that inorganic nitrate can reduce BP in both healthy subjects and hypertensive patients, presumably via the alternative pathway (Kapil et al., 2010, 2015). However, due to the transient nature of nitrite and NO, their concentrations may not accurately reflect metabolic turnover, which could explain the stable plasma nitrites levels observed in our study. Interestingly, an experimental study by Carlström et al. subjected Sprague–Dawley rats to a chronic high sodium diet from 3 weeks of age. The rats developed hypertension, renal fibrosis, proteinuria, and other signs of renal damage. However, adding a simultaneous inorganic nitrate supplementation to their diet decreased hypertension dose‐dependently and almost prevented proteinuria and signs of renal damage (Carlström et al., 2011). Carlström et al. found that the nitrate supplementation was associated with a reduced level of oxidative stress, which they attributed to scavenging of O^2−^ by nitrate‐derived NO through activation of the alternative pathway (Carlström et al., 2011, 2015). These findings support the possibility that our results reflect an increased activation of the alternative pathway, leading to greater utilization of nitrate for the generation of nitrate‐derived NO. Our observations highlight the importance of understanding nitrate metabolism beyond classical pathways and suggest that sodium intake may influence systemic nitrate utilization. However, further research is needed to elucidate the underlying mechanisms, including detailed investigation of the specific renal handling of nitrate and nitrite, as well as clinical studies in larger cohorts to clarify potential clinical implications, particularly in sodium‐sensitive groups such as individuals with hypertension or heart failure.

In our study, sex‐specific subgroup analysis revealed that females exhibited higher plasma nitrate levels during low sodium intake, as well as a more pronounced reduction in plasma nitrate following high sodium intake. Furthermore, urinary nitrate excretion showed a sex‐related difference in response to sodium intake: a decrease in females, whereas levels remained stable in males. The observed interaction effects between sodium intake and sex for both plasma nitrate and urinary nitrate excretion further support a potential sex‐specific response to sodium intake in nitrate handling. In line with previous findings by Sundqvist and Kapil, our data also suggest that the physiological handling of nitrate may be influenced by sex‐related factors (Kapil et al., 2018; Sundqvist et al., 2021). Estrogen has previously been shown to affect endothelial nitric oxide synthase (eNOS) activity and thereby enhance NO formation (Chambliss & Shaul, 2002; Ray et al., 2007). Interestingly, the pronounced reduction in plasma nitrate observed among females appeared to account for the overall decline in plasma nitrate levels within the total cohort during high sodium intake. Further highlighting sex‐related differences in nitrate handling, we observed significantly lower urinary nitrate excretion in females following high sodium intake. This finding, together with results from the full cohort, suggests that the greater utilization of nitrate under high sodium conditions is likely driven by the female response. These observations may generate new hypotheses—for instance, that females may be better protected against salt‐sensitive hypertension due to the protective effects of estrogen, including enhanced eNOS activation and maybe an increased engagement of the alternative pathway (Bailey & Dhaun, 2024; Brinson et al., 2014; Chambliss & Shaul, 2002; Iorga et al., 2017; O'Donnell et al., 2014; Pilic et al., 2016; Visniauskas et al., 2023).

Regarding nitrite, we found no differences in plasma concentrations, renal clearance, or fractional excretion between the high and low sodium intake. Nitrite is a considerably more transient compound than nitrate, and its further endogenous reduction to NO involves multiple complex enzymatic and nonenzymatic pathways. Moreover, nitrite appears in much lower concentrations in both plasma and urine compared to nitrate, which may make it more challenging to detect changes in response to physiological stimuli (Lundberg et al., 2009). However, we did observe that changes in BP in response to sodium intake correlated positively with plasma nitrite levels. This finding aligns with previous studies demonstrating associations between plasma nitrite and hemodynamic parameters, including endothelial function and BP regulation (Kapil et al., 2018; Kleinbongard et al., 2006). For example, Kapil et al. (2018) reported an inverse correlation between plasma nitrite and systolic BP, suggesting that higher nitrite levels were associated with lower systolic BP. In contrast, our study found a positive correlation between changes in plasma nitrite and changes in both systolic and diastolic BP. Although we did not replicate the inverse relationship described by Kapil et al., our findings support the growing body of evidence linking plasma nitrite to BP regulation. Since plasma nitrite is considered a marker of eNOS activity, the observed correlations in our study may reflect increased shear stress induced by high sodium intake, potentially triggering a compensatory endothelial response (Boegehold, 2013; Kelm et al., 1999; Kleinbongard et al., 2006). Overall, while the direction of the association may differ, our results reinforce the physiological relevance of plasma nitrite in human BP regulation (Kapil et al., 2010, 2013, 2015, 2018).

### 4.1 Strength and limitations

Our study was designed as a randomized, placebo‐controlled, double‐blinded, crossover trial, which is a major strength supporting the validity of our findings.

The study was conducted with a strict control of dietary intake, including standardized levels of nitrate, nitrite, sodium, and potassium prior to the examination days. As dietary intake contributes substantially to both plasma and urinary nitrate/nitrite levels, this control is important. In humans, an estimated >80% of nitrate intake stems from vegetables and other foods, while only ~15%–20% originates from drinking water (Lundberg et al., 2006). In humans, only a small amount of NO is produced endogenously via NOS, estimated to roughly 1 mmol/day (Castillo et al., 1996; Sakinis et al., 1999). Therefore, studies relying on plasma and urinary nitrate/nitrite measures as markers of NO formation are highly influenced by diet. To mitigate this, subjects followed a 4‐day standardized diet during both the low and high sodium phase, which strengthens our results. Adherence to the allocated sodium intake was verified by increased urinary sodium excretion and plasma sodium levels during high sodium intake. However, it cannot be ruled out that dietary changes may alter the microbiome of the oral cavity, thereby affecting the endogenous reduction of nitrate to nitrite.

As this was a post hoc analysis of additional secondary outcome measures, limitations include a probable risk of type 1 and 2 errors, especially when subgrouping by sex, as these analyses may be underpowered.

## 5 CONCLUSION

In conclusion, our results demonstrated a decrease in plasma nitrate after increased sodium intake that could not be explained by increased renal excretion, particularly in women. These findings suggest enhanced systemic utilization of nitrate as a potential adaptive mechanism to sodium loading. Notably, significant sex and interaction effects observed in plasma and urinary nitrate support the presence of sex‐specific responses in nitrate handling during sodium intake. Given the emerging role of nitrate/nitrite/NO pathways in cardiovascular and renal physiology, these observations may have broader implications for understanding sodium homeostasis and sex‐specific adaptations. Future research should aim to clarify the renal and systemic utilization of nitrate/nitrite, especially at the tubular level, and explore potential clinical relevance for sodium‐sensitive conditions such as hypertension, chronic kidney disease, and heart failure.

## AUTHOR CONTRIBUTIONS

All authors have contributed to the publication. JNB, RLS, and AMØ designed the project. RLS, AMØ, and MHV performed the experiments and laboratory analyses. AMØ performed the statistical analyses and drafted the manuscript. AMØ, MHV, JBR, FHM, and JNB edited the manuscript. All authors read and approved the final manuscript.

## FUNDING INFORMATION

Support was provided solely from institutional and departmental sources.

## CONFLICT OF INTEREST STATEMENT

The authors declare no competing interests.

## ETHICS STATEMENT

This study was approved by the Regional Committee on Biomedical Research Ethics (case number: 1‐10‐72‐351‐15). Informed, signed consent was obtained from each patient. The study was carried out in accordance with the Declaration of Helsinki.

## Supporting information


Appendix S1.

## Data Availability

The datasets used and analyzed during the current study are available from the corresponding author on reasonable request.
