# Supplementary material for: Effect of sodium balance on levels of nitrates in healthy subjects: Posthoc analysis of a randomized, double‐blinded, placebo‐controlled, crossover study
Source: Physiol Rep. 2025 Aug 19;13(16):e70512. doi: 10.14814/phy2.70512 (PMC12361809; doi:10.14814/phy2.70512)
Supplement: Supplementary file 1 — Appendix S1. [file PHY2-13-e70512-s001.docx]

***Supplementary materials.***

| **Table S1.** GFR and tubular function (n=27) | | | |
| --- | --- | --- | --- |
| **Variable** | **Low sodium** | **High sodium** | ***P*-value** |
| **GFR (mL/min/1.73m^2^)** | 87 ± 14 | 91 ± 14 | 0.051 |
| UNa (mmol/24 hours) | 101 ± 42 | 286 ± 58 | <0.001 |
| **UK (mmol/24 hours)** | 66 ± 18 | 67 ± 16 | 0.921 |
| **FE_Na_ (%)** | 0.37 ± 0.17 | 0.99 ± 0.21 | <0.001 |
| **Urine output (mL/min)** | 7.7 ± 3.4 | 7.9 ± 2.9 | 0.745 |
| Glomerular filtration rate from ^51^Cr-EDTA clearance (GFR), absolute urinary excretion of sodium (U-Na) and potassium (U-K), fractional excretion of sodium (FE_Na_) and urine output. Values are shown as means ± SD in brackets or medians with 25 and 75 percentiles in brackets. Statistics are performed with a paired *t*-test or Wilcoxon signed rank test to test difference in response between diets. | | | |

| **Table S2.** Hemodynamic variables (n=27) | | | |
| --- | --- | --- | --- |
| **Variable** | **Low sodium** | **High sodium** | ***P*-value** |
| **Systolic blood pressure (mmHg)** | 114 ± 7 | 116 ± 10 | 0.174 |
| **Diastolic blood pressure (mmHg)** | 67 ± 6 | 67 ± 7 | 0.441 |
| **Heart rate (beats pr. min)** | 56 ± 8 | 55 ± 9 | 0.492 |
| **PVW** | 5.1 ± 0.4 | 5.1 ± 0.5 | 0.132 |
| Brachial systolic and diastolic blood pressure (SBP, DBP), heart rate (HR), and pulse wave velocity (PWV). Values are shown as means ± SD in brackets or medians with 25 and 75 percentiles in brackets. Statistics are performed with a paired *t*-test or Wilcoxon signed rank test to test difference in response between diets. | | | |

| **Table S3.** Hemodynamic variables divided by sex. | | | |
| --- | --- | --- | --- |
| *Hemodynamic variables in males (n=14)* | | | |
| **Variable** | **Low sodium** | **High sodium** | ***P*-value** |
| **GFR (mL/min/1.73m^2^)** | 84 ± 14 | 87 ± 14 | 0.517 |
| **Systolic blood pressure (mmHg)** | 117 ± 7 | 120 ± 11 | 0.662 |
| **Diastolic blood pressure (mmHg)** | 69 ± 5 | 69 ± 7 | 0.973 |
|  | | | |
| *Hemodynamic variables in females (n=13)* | | | |
| **GFR (mL/min/1.73m^2^)** | 91 ± 14 | 94 ± 13 | 0.598 |
| **Systolic blood pressure (mmHg)** | 111 ± 6 | 113 ± 8 | 0.485 |
| **Diastolic blood pressure (mmHg)** | 64 ± 5 | 67 ± 7 | 0.529 |
| Glomerular filtration rate from ^51^Cr-EDTA clearance (GFR), brachial systolic and diastolic blood pressure (SBP, DBP). Values are shown as means ± SD in brackets or medians with 25 and 75 percentiles in brackets. Statistics are performed with a unpaired *t*-test or Mann Whitney test to test difference in response between sex. | | | |

| **Table S4.** The nitric oxide system divided by sex. | | | |
| --- | --- | --- | --- |
| *The nitric oxide system in males (n=14)* | | | |
| **Variable** | **Low sodium** | **High sodium** | ***P*-value** |
| **p-Nitrate (µmol/L)** | 20[15;24] | 17[14;28] | 0.720 |
| **p-Nitrite (µmol/L)** | 0.17 ± .04 | 0.19 ± 0.06 | 0.177 |
| **p-cGMP (pmol/mL)** | 115 ± 21 | 107 ± 23 | 0.239 |
| **U-Nitrate (µmol/min)** | 0.86[0.52;1.11] | 0.77[0.61;1.37] | 0.541 |
| **U-Nitrite** **(nmol/min)** | 1.3[0.06;1.5] | 1.5[1.1;1.9] | 0.164 |
| **Nitrate clearance (mL/min)** | 40[35;44] | 46[38;51] | 0.155 |
| **Nitrite clearance (mL/min)** | 7[3;9] | 8[5;13] | 0.635 |
| **Nitrate clearance (mL/min/m^2^)** | 19[17;22] | 22[18;25] | 0.155 |
| **Nitrite clearance (mL/min/m^2^)** | 3[2;4] | 3[2;6] | 0.571 |
| **FE_Nitrate_ (%)** | 43[40;54] | 47[45;53] | 0.128 |
| **FE_Nitrite_ (%)** | 9.0[4.1;10.6] | 9.2[5.6;13.9] | 0.571 |
|  | | | |
| *The nitric oxide system in females (n=13)* | | | |
| **p-Nitrate (µmol/L)** | 29[24;43] | 15[11;26] | 0.003 |
| **p-Nitrite (µmol/L)** | 0.23 ± 0.05 | 0.25 ± 0.04 | 0.338 |
| **p-cGMP (pmol/mL)** | 86 ± 24 | 95 ± 20 | 0.333 |
| **U-Nitrate (µmol/min)** | 1.05[0.65;1.77] | 0.57[0.39;0.98] | 0.022 |
| **U-Nitrite** **(nmol/min)** | 1.7[1.3;2.0] | 1.3[1.1;3.1] | 0.960 |
| **Nitrate clearance (mL/min)** | 34[29;39] | 35[32;44] | 0.362 |
| **Nitrite clearance (mL/min)** | 7[5;9] | 5[4;11] | 0.687 |
| **Nitrate clearance (mL/min/m^2^)** | 19[16;21] | 20[18;24] | 0.362 |
| **Nitrite clearance (mL/min/m^2^)** | 4[3;5] | 4[3;6] | 0.650 |
| **FE_Nitrate_ (%)** | 39[31;45] | 40[36;45] | 0.687 |
| **FE_Nitrite_ (%)** | 8.3[6.4;10.2] | 5.4[4.2;13.7] | 0.687 |
| Plasma concentrations of nitrate, nitrite and cyclic guanosine monophosphate (cGMP). Urinary excretion rate of nitrate (nitrate/minute), nitrite (nitrite/minute). Renal clearance of nitrate and nitrite (mL/minute), fractional excretion of nitrate (FE_Nitrate_) and nitrite (FE_Nitrite_). Values are shown as means ± SD in brackets or medians with 25 and 75 percentiles in brackets. Statistics are performed with a unpaired *t*-test or Mann Whitney test to test difference in response between sex. | | | |

| Table S5. | | | | | | | | | |
| --- | --- | --- | --- | --- | --- | --- | --- | --- | --- |
| Two-way ANOVA results for all outcome variables. | | | | | | | | | |
| Variable | **F (Interv.)** | **P**  **(Interv.)** | **η² (Interv.)** | **F**  **(Sex)** | **P**  **(Sex)** | **η² (Sex)** | **F**  **(Interv.×Sex)** | **P (Interv.×Sex)** | **η² (Interv.×Sex)** |
| GFR | 0.404 | 0.404 | 0.014 | 3.906 | 0.054 | 0.072 | 0.006 | 0.937 | 0.000 |
| UNa | 184.2 | 0.000 | 0.787 | 3.183 | 0.080 | 0.060 | 0.011 | 0.918 | 0.000 |
| UK | 0.011 | 0.915 | 0.000 | 2.798 | 0.101 | 0.053 | 0.433 | 0.514 | 0.009 |
| Urine output | 0.065 | 0.800 | 0.001 | 2.825 | 0.099 | 0.053 | 0.592 | 0.445 | 0.012 |
| Systolic BP | 0.609 | 0.439 | 0.012 | 6.870 | 0.012 | 0.121 | 0.010 | 0.922 | 0.000 |
| Diastolic BP | 0.196 | 0.660 | 0.004 | 5.506 | 0.023 | 0.099 | 0.239 | 0.627 | 0.005 |
| Heart rate | 0.148 | 0.702 | 0.003 | 6.224 | 0.016 | 0.111 | 1.102 | 0.299 | 0.022 |
| PWV | 0.330 | 0.568 | 0.007 | 3.394 | 0.071 | 0.064 | 0.007 | 0.935 | 0.000 |
| p-Nitrate | 5.390 | 0.025 | 0.099 | 3.162 | 0.082 | 0.061 | 6.914 | 0.011 | 0.124 |
| p-Nitrite | 2.763 | 0.102 | 0.053 | 0.075 | 0.785 | 0.001 | 0.000 | 0.987 | 0.000 |
| p-cGMP | 0.015 | 0.903 | 0.000 | 10.778 | 0.002 | 0.177 | 2.397 | 0.128 | 0.046 |
| p-nitrate/nitrite | 0.021 | 0.021 | 0.021 | 1.165 | 0.287 | 0.022 | 3.156 | 0.082 | 0.061 |
| UV-Nitrate | 1.924 | 0.172 | 0.037 | 0.010 | 0.922 | 0.000 | 6.113 | 0.017 | 0.109 |
| UV-Nitrite | 1.366 | 0.248 | 0.027 | 6.394 | 0.015 | 0.113 | 0.159 | 0.692 | 0.003 |
| Nitrate Cl | 0.722 | 0.722 | 0.001 | 0.002 | 0.993 | 0.000 | 0.121 | 0.728 | 0.002 |
| Nitrite Cl | 0.380 | 0.380 | 0.007 | 0.020 | 0.838 | 0.001 | 0.046 | 0.830 | 0.001 |
| Nitrate Cl (BSA) | 0.730 | 0.730 | 0.001 | 0.379 | 0.379 | 0.007 | 0.088 | 0.768 | 0.002 |
| Nitrite Cl (BSA) | 0.768 | 0.768 | 0.002 | 0.387 | 0.387 | 0.008 | 0.088 | 0.768 | 0.002 |
| FENitrate | 0.404 | 0.921 | 0.008 | 8.875 | 0.004 | 0.153 | 0.046 | 0.830 | 0.001 |
| FENitrite | 0.991 | 0.991 | 0.000 | 0.085 | 0.767 | 0.002 | 0.020 | 0.888 | 0.000 |
| U-nitrate/nitrite | 0.244 | 0.244 | 0.001 | 2.825 | 0.035 | 0.053 | 0.061 | 0.807 | 0.001 |
| The two-way-ANOVA results, summarizing the effects of intervention (low vs. high sodium intake), sex, and their interaction (intervention × sex) on all measured variables. Values are shown as F-statistics, corresponding p-values, and partial eta squared (η²ₚ) as effect size estimates. Statistical significance was defined as p < 0.05. | | | | | | | | | |

**Figure S1:**

The absolute changes from high to low sodium intake (Table 3) showed a weak positive correlation between plasma nitrite and systolic BP (Spearman's correlation, R^2^ = 0.148, P = 0.047).

**Figure S2:**

The absolute changes from high to low sodium intake (Table 3) showed a weak positive correlation between plasma nitrite and diastolic BP (Spearman's correlation, R^2^ = 0.228, P = 0.012).
